# Peripheral T‐Cell Lymphoma: What's Next?

**DOI:** 10.1002/hon.70069

**Published:** 2025-06-15

**Authors:** Sang Eun Yoon, Won Seog Kim

**Affiliations:** ^1^ Division of Hematology‐Oncology Department of Medicine Samsung Medical Center Sungkyunkwan University School of Medicine Seoul South Korea

**Keywords:** brentuximab‐vedotin, CHOP, follicular helper T‐cell lymphoma, peripheral T‐cell lymphoma

## Abstract

Peripheral T‐cell lymphoma (PTCL) is a rare and heterogeneous group of diseases, with over 30 subtypes according to the International Consensus Classification of Mature Lymphoid Neoplasms (ICC) and World Health Organization Classification of Hematolymphoid Tumors (WHO‐HEM) 2022. The classification complexity reflects the underlying genetic and biological diversity of PTCL. For decades, distinct PTCL subtypes have been uniformly treated with CHOP (cyclophosphamide, doxorubicin, vincristine, and prednisone) or CHOP‐like regimens originally developed for mainly B‐cell lymphoma. Attempts to improve frontline CHOP‐plus strategies have failed mainly due to toxicities and lack of biological rationale. Only the ECHELON‐2 trial succeeded as more than 70% of patients had anaplastic large cell lymphoma (ALCL), where brentuximab vedotin (BV) is most effective. Looking ahead to 2025 and beyond, future treatment strategies for PTCL should be guided by a deeper understanding of its underlying biology rather than relying on empirical extrapolations from other lymphomas.

## 1 Introduction

### 1.1 PTCL

PTCL is a rare and heterogeneous disease with a poor prognosis, characterized by significant genetic and biological diversity [1, 2]. Contemporary classification systems, including the ICC and WHO‐HEM 2022, categorize PTCL based on cell of origin, genetic alterations, and biological characteristics [3, 4]. These refined classifications provide a better framework for guiding treatment strategies, particularly for incorporating biologically targeted therapies. Traditional treatments, such as CHOP‐based regimens, have shown limited efficacy due to the diverse nature of PTCL subtypes [5, 6]. A biologically driven approach is now essential for improving patient outcomes. This review will focus on the treatment strategies for nodal PTCL, emphasizing recent advances and the potential role of targeted therapies. This reliance primarily stems from the absence of a superior multi‐agent chemotherapy regimen and historical precedence.

### 1.2 Frontline Treatment

CHOP remains the most widely used frontline regimen for PTCL despite its limitations. This reliance primarily stems from the absence of a superior multi‐agent chemotherapy regimen and historical precedence [2, 8]. The pivotal phase III trial by Fisher et al. established CHOP as the standard for aggressive lymphomas, but the study was conducted primarily in B‐cell lymphoma patients, not PTCL [7]. Consequently, CHOP was extrapolated to PTCL treatment, although its efficacy in these genetically and biologically diverse subtypes remains suboptimal. However, CHOP has failed to achieve satisfactory long‐term outcomes in PTCL, with low overall survival rates and high relapse rates. However, the lack of large‐scale randomized trials specific to PTCL has left clinicians with limited alternative options. As a result, many still consider CHOP the default regimen, though it is unlikely to be the optimal approach.

Furthermore, for several rare PTCL subtypes—including extranodal NK/T‐cell lymphoma (ENKTL), monomorphic epitheliotropic intestinal T‐cell lymphoma (MEITL), and hepatosplenic T‐cell lymphoma (HSTCL)—CHOP is not recommended as standard care due to poor efficacy [9, 10, 11]. These subtypes require biologically tailored therapies, reflecting the need for a paradigm shift in PTCL treatment. Future strategies should focus on biologically driven approaches rather than continuing reliance on CHOP, which was never designed for PTCL's heterogeneous disease spectrum.

## 2 CHOP‐Plus: Challenges and Future Directions

Several CHOP‐plus trials have not demonstrated meaningful survival benefits for two reasons. First, the lack of biologically driven patient selection resulted in the enrollment of a broad spectrum of PTCL patients without considering their genetic or molecular heterogeneity, thereby diluting the potential benefits of the added agents. Second, the dose intensity of the novel agents was often compromised to maintain the standard CHOP regimen. As a result, these regimens functioned more like CHOP‐minus rather than proper CHOP‐plus strategies, limiting their overall effectiveness (Table 1).

**TABLE 1:** Clinical study overview of CHOP plus additional agent.

Experimental regimen	Trial design	Numbers	Outcomes
Romidepsin‐CHOP [12]	Phase III	421	CR 41%; 2Y PFS 43%
Alemtuzumab‐CHOP [13]	Phase III	136 (age 18–65)	CR 52%; 3Y PFS 37%
Lenalidomide‐CHOP [14]	Phase III	116 (age 61–80)	CR 60%; 3Y PFS 28%
Lenalidomide‐CHOP [15]	Phase II	80	CR 41%; 2Y PFS 42%
Lenalidomide‐CHOEP [16]	Phase II	39	CR 49%; 2Y PFS 55%
Denileukin diftitox‐CHOP [17]	Phase II	41	CR 55%; 2Y PFS 43%
Vorinostat‐CHOP [18]	Phase I	14	CR 12/14 (93%)
Belinostat‐CHOP [19]	Phase I	32	CR10/14 (71%) at the MTD
Chidamide‐CHOP [20]	Phase I	30	CR 25/28 (89%)
Chidamide‐CHOEP [21]	Phase Ib/II	142	CR 40%; 2Y PFS 38%
Azacytidine‐CHOP [22]	Phase II	20	CR 75%; 2Y PFS 65%
Everolimus‐CHOP [23]	Phase II	30	CR 56%; 2Y PFS 33%
Pralatrexate‐CHOP [24]	Phase I	52	CR 66%
Pralatrexate‐CEOP [25]	Phase II	33	CR 50%; 2Y PFS 39%
Bortezomib‐CHOP [26]	Phase II	46	CR 65%, 3Y PFS 35%
Bevacizumab‐CHOP [27]	Phase II	39	CR 49%; 1Y PFS 44%

Abbreviations: CR, complete response; MTD, maximum tolerated dose; PFS, progression free‐survival; Y, year.

## 3 ECHELON‐2 Trial: Key Factors Behind Its Success

The ECHELON‐2 trial is a notable exception among CHOP‐plus studies, as it demonstrated significant clinical benefits. One key factor in its success was the selective enrollment of ALCL patients, with over 70% of participants diagnosed with ALCL. Brentuximab vedotin (BV), an antibody‐drug conjugate targeting CD30, is most effective in ALCL, so this patient selection maximized the trial's therapeutic impact [7]. Another advantage of the ECHELON‐2 trial was optimized dose intensity. Unlike other CHOP‐plus regimens, vincristine was omitted, allowing BV to be administrated at its full intended dose without compromising efficacy. As a result, the trial achieved superior outcomes compared to standard CHOP therapy. The key findings from ECHELON‐2 included a significantly higher overall response rate (ORR) of 82% versus 72% (*p* = 0.0032) and a complete response rate (CRR) of 68% versus 56% (*p* = 0.0066) in BV‐CHOP versus CHOP. The study also demonstrated significant improvements in progression‐free survival (PFS) and overall survival (OS). Subgroup analysis revealed the most significant PFS benefit in ALK‐positive ALCL, followed by ALK‐negative ALCL, whereas the benefit was more limited in PTCL‐NOS and angioimmunoblastic T‐cell lymphoma (AITL). These results highlight the importance of biologically driven patient selection and dose optimization in improving treatment outcomes for PTCL.

## 4 Romidepsin‐CHOP Versus CHOP: Lack of Significant Benefit

The romidepsin‐CHOP (romi‐CHOP) trial failed to demonstrate a significant advantage over standard CHOP therapy in terms of PFS and OS [28]. Despite the addition of romidepsin, outcomes remained comparable between the two treatment arms, suggesting that romidepsin did not provide sustained benefit when combined with CHOP. However, an exploratory analysis revealed a potential benefit in a specific subgroup. In follicular helper T‐cell lymphoma (TFH) cases, the median PFS was significantly longer in the Romi‐CHOP arm (19.5 vs. 10.6 months; HR 0.703, *p* = 0.039). Given that the histological composition of the trial reflected epidemiological distributions, with AITL representing approximately 47% of cases, the potential impact of a higher proportion of AITL patients remains unclear. This highlights the potential need for more refined, biology‐driven patient selection in future studies.

## 5 Future Directions: Genomics‐Based CHOP‐Plus Regimens

The failure of CHOP‐plus strategies emphasizes the necessity for a genomics‐based approach in PTCL treatment. Unlike traditional chemotherapy regimens that treat all PTCL subtypes as a homogeneous group, a genomics‐driven strategy could optimize treatment selection based on tumor biology. By identifying the molecular characteristics of each subtype, treatments can be tailored to enhance efficacy and improve patient outcomes.

Future strategies should focus on several key areas. First, stratifying patients based on generic and molecular markers can help match them with the most effective targeted therapies. Second, developing CHOP modifications tailored to specific PTCL subtypes rather than applying a one‐size‐fits‐all approach may lead to better treatment precision. Third, incorporating novel targeted agents, such as epigenetic modulators and immunotherapies, can enhance treatment efficacy and improve survival outcomes [29].

By integrating genomic insights, future CHOP‐plus regimens may overcome the limitations of current strategies and provide more effective, personalized treatments for PTCL. This approach can increase response rates, prolong survival, and improve overall patient outcomes in this heterogeneous and challenging disease. The failure of traditional CHOP‐plus trials highlights the urgent need for precision medicine in PTCL. Future approaches should prioritize biologically driven patient selection and targeted modifications of CHOP regimens to achieve meaningful survival improvements.

## 6 Upfront Autologous Hematopoietic Stem Cell Transplantation (Auto‐HSCT)

The role of upfront auto‐HSCT in PTCL remains controversial, as selection bias cannot be eliminated. While retrospective studies suggest potential benefits, definitive evidence is lacking. The two ongoing randomized trials are investigating the efficacy of upfront auto‐HSCT in PTCL patients achieving complete remission (CR). The TRANSCRIPT study evaluates induction chemotherapy alone (Group A) or induction chemotherapy followed by consolidation with auto‐HSCT (Group B) in patients with nodal PTCL to determine whether auto‐HSCT improves long‐term outcomes. The JCOG2210, TRANSFER study is being conducted in Japan to assess the effectiveness of auto‐HSCT in PTCL and clarify its role in standard treatment strategies [30]. These trials are expected to provide crucial data to help define the optimal use of upfront auto‐HSCT in PTCL, potentially guiding future treatment recommendations and improving patient outcomes.

## 7 Allogenic Hematopoietic Stem Cell Transplantation (Allo‐HSCT)

The role of allo‐HSCT in PTCL remains debated. Studies comparing auto‐HSCT and allo‐HSCT have shown no significant differences in OS. However, relapse‐related mortality is higher in auto‐HSCT, whereas non‐relapse‐related mortality (NRM) is higher in allo‐HSCT, primarily due to graft‐versus‐host disease (GVHD) and transplant‐related complications. Despite its risks, allo‐HSCT offers the potential for long‐term remission through the graft‐versus‐lymphoma (GVL) effect. Further research is needed to identify the optimal candidates for allo‐HSCT and improve transplant‐related outcomes in PTCL [31].

## 8 Chemotherapy‐Free Regimens in PTCL: Emerging Strategies

Chemotherapy‐free regimens have gained interest in PTCL due to their potential to improve efficacy while reducing toxicity. However, data on chemotherapy‐free strategies remain limited. One promising approach is the combination of 5‐azacitidine (5‐aza) and romidepsin (Romi), which has shown encouraging results in clinical trials [32]. In all T‐cell lymphoma patients, the ORR was 61%, with a CR rate of 43%. The response was even higher in FHT‐lymphoma, with an ORR of 80% and a CR rate of 67%. Notably, in treatment‐naïve T‐cell lymphoma patients, the ORR and CR rates were 70% and 50%, respectively. Given these findings, a chemotherapy‐free regimen may be a superior option for treatment‐naïve FHT lymphoma patients, as their ORR and CR rates surpass those observed with conventional chemotherapy‐based regimens. While further validation is needed, these results highlight the potential role of epigenetic and targeted therapies in PTCL, paving the way for novel frontline or relapsed/refractory treatment strategies beyond standard chemotherapy.

## 9 Conclusion

With the growing availability of genomic and biological data, further treatment strategies for PTCL should be developed based on current molecular information. Advances in targeted therapies, genomics‐based CHOP modifications, and chemotherapy‐free regimens offer promising alternatives to traditional chemotherapy. However, significant challenges remain, including rarity, heterogeneity, and aggressive clinical behavior of PTCL, which complicate treatment optimization. While novel agents, biologically driven patient selection, and precision medicine approaches hold great potential, further clinical trials are needed to refine treatment strategies and improve patient outcomes in this complex and diverse disease.

## Conflicts of Interest

The authors declare no conflicts of interest.

## Peer Review

The peer review history for this article is available at https://www.webofscience.com/api/gateway/wos/peer-review/10.1002/hon.70069.

## Data Availability

All data will become publicly available upon request to the corresponding authors.
